# Optical observations of embolism in three conifers overestimate the vulnerability of stem xylem to hydraulic dysfunction

**DOI:** 10.1093/jxb/eraf075

**Published:** 2025-02-28

**Authors:** Adriano Losso, Feng Feng, Barbara Beikircher, Stefan Mayr

**Affiliations:** Department of Botany, Universität Innsbruck/University of Innsbruck, Innsbruck, Austria; Department of Botany, Universität Innsbruck/University of Innsbruck, Innsbruck, Austria; Department of Botany, Universität Innsbruck/University of Innsbruck, Innsbruck, Austria; Department of Botany, Universität Innsbruck/University of Innsbruck, Innsbruck, Austria; Ghent University, Belgium

**Keywords:** Acoustic emissions, conifers, optical technique, vulnerability to drought, xylem, xylem embolism

## Abstract

Hydraulic failure due to drought stress is a major cause of forest decline. Many techniques have been developed to test the vulnerability of trees to drought-induced xylem embolism, each with advantages and limitations. We quantified drought vulnerability using optical vulnerability and ultrasonic acoustic emission (UAE) techniques by performing simultaneous measurements on branches of three conifers (*Picea abies*, *Pinus sylvestris*, and *Pinus cembra*). Results were compared with vulnerability curves obtained using the flow–centrifuge (FC) technique. With respect to the optical vulnerability method, the light transmission properties of the samples were analyzed to determine the xylem fraction observed. Optical vulnerability thresholds were 0.3–1.4 MPa higher than those obtained with UAE and FC techniques, which were similar overall. Xylem depths observed by optical vulnerability were limited to the peripheral 0.15–0.20 mm, as no light transmission was detected at greater depth. Light transmission was higher in saturated xylem than in dry. The results indicatet that xylem embolism detection by optical vulnerability is limited to thin, peripheral xylem layers, and that vulnerability thresholds may differ from those of thicker samples (>0.4 mm) measured with UAE and FC techniques. Therefore, optical vulnerability may not accurately reflect the general vulnerability of thick branches, as only peripheral xylem layers are analyzed. Changes in light transmission during dehydration must be considered.

## Teaser

Optical visualization of xylem embolism is limited to thin, peripheral xylem layers, and vulnerability thresholds may differ from those of thicker samples. Light transmission was observed to decrease in dry xylem.

## Introduction

In trees, water is transported in the xylem network under negative pressures, which are created in the leaves by transpiration (i.e. low water potential, Ψ) and are transmitted down to the roots where water uptake takes place. According to the cohesion–tension theory, water in the xylem is in a so-called metastable state (Tyree and Zimmermann, 2002) and thus at risk of transition from liquid to vapor (Cochard, 2006). This transition leads to the formation of embolisms that can spread throughout the xylem network, reducing hydraulic conductance and therefore plant productivity, and in extreme cases even causing plant death (Sperry *et al.*, 1993; Brodribb and Cochard, 2009).

Drought stress is known to be the main cause of xylem embolism, which is initiated when the tension in the sap causes gas bubbles to be aspirated through the pits into adjacent xylem conduits (air seeding; Tyree and Zimmermann, 2002). The vulnerability of xylem to drought-induced embolism can be quantified by vulnerability curves, used to describe the progressive impairment of the hydraulic network as a function of Ψ (e.g. Sperry *et al.*, 1988). Over the past decades, several methods have been developed to construct vulnerability curves on different plant species as well as plant organs (e.g. Sperry *et al.*, 1988; Brodribb and Holbrook, 2006; Hietz *et al.*, 2008; Brodribb and Cochard, 2009; Brodersen *et al.*, 2010; Losso *et al.*, 2016, 2019; Pereira *et al.*, 2016; Rodriguez-Dominguez *et al.*, 2018; Feng *et al.*, 2021). Among these methods, vulnerability curves can be constructed by using centrifugal force to generate a certain Ψ in the xylem via spinning stem samples and subsequently (Alder *et al.*, 1997) or simultaneously measuring the flow [i.e. flow–centrifuge (FC) technique; Beikircher *et al.*, 2010; Cochard *et al.*, 2013], by recording ultrasonic acoustic emissions (UAEs) as they are emitted on air seeding when samples dry (Tyree and Sperry, 1989; Ponomarenko *et al.*, 2014; Nolf *et al.*, 2015), or by acquiring images of xylem embolism-induced changes under light transmission (optical visualization, OV; Brodribb *et al.*, 2016, 2017) in dehydrating samples. Other important methods include hydraulic measurements (Sperry method; Sperry *et al.*, 1988) on previously dehydrated samples, staining approaches (Hietz *et al.*, 2008; Nolf *et al.*, 2016), and X-ray microtomography aimed at quantifying changes in water-filled xylem area during progressive dehydration of samples (e.g. Brodersen *et al.*, 2010; Nardini *et al.*, 2017; Losso *et al.*, 2019). All of these methods have advantages and limitations that have been discussed in numerous studies (e.g. Cai and Tyree, 2010; Cochard *et al.*, 2013; Venturas *et al.*, 2019; Sergent *et al.*, 2020). However, a shared advantage of the FC, UAE, and OV techniques is that a complete vulnerability curve can be generated from a single plant organ (e.g. branch), thus requiring little plant material and enabling estimation of variability across samples. UAE and OV can also be used simultaneously on the same plant (Shelden *et al.*, 2017; Rodriguez-Dominguez *et al.*, 2018; Peters *et al.*, 2020).

The FC technique requires perfectly straight samples with a length corresponding to the size of the centrifuge rotor used. During spinning, embolism formation is induced in the center of the sample (but see also Silva *et al.*, 2024), but is quantified in the entire sample. The main limitation of this method is the potential for open vessel artifacts, which can cause premature embolization (e.g. Cochard *et al.*, 2013; Torres-Ruiz *et al.*, 2017; López *et al.*, 2018) and thus overestimate xylem vulnerability to embolism. In conifers, the small tracheids (length <6 mm; Sperry *et al.*, 2006) do not cause open vessel artifacts; however, when small rotors are used, pit aspiration can considerably reduce the total conductance measured when spinning the samples (Beikircher *et al.*, 2010). In addition, measurements can be difficult when resin clogs the xylem.

UAE is a non-destructive and non-invasive method that can be used to record embolism formation (Ponomarenko *et al.*, 2014) in plants and plant organs (Rosner *et al.*, 2006; Vergeynst *et al.*, 2015; Charrier *et al.*, 2017). Acoustic events can exceed the theoretical number of conducting conduits in the xylem (Rosner *et al.*, 2006; Kasuga *et al.*, 2015; Vergeynst *et al.*, 2015), but the relative course of cumulative events often correlates well with the hydraulically measured vulnerability curves, especially in conifers. In angiosperms, acoustic signals are often recorded after xylem conduits have been completely embolized, possibly as a result of embolism events in fiber cells (Wolkerstorfer *et al.*, 2012; Nolf *et al.*, 2015), which do not contribute to the total hydraulic conductance.

Optical visualization is also non-destructive and is the most recently developed of the three methods (Brodribb *et al.*, 2016, 2017). It is a relatively inexpensive alternative to build vulnerability curves, allowing a wide range of equipment to be used to provide reliable images of changes in light transmission caused by xylem embolism (Hochberg *et al.*, 2017; Cardoso *et al.*, 2018; Johnson *et al.*, 2020; Petruzzellis *et al.*, 2020). Images enable a direct quantification of embolized xylem area, and can be used across different organs, providing both high spatial and temporal resolution (e.g. Cardoso *et al.*, 2022). However, one aspect of the OV method (and somehow neglected in the past) is the xylem depth to which light transmission is limited. In this study, we thus tested for the light transmission properties of the samples by measuring the amount of light passing through progressively thicker xylem sections to estimate the xylem fraction observed by OV.

In recent years, a number of studies have compared vulnerability curves obtained with different methods, also including the new OV, showing in some cases higher and in others lower agreement with the FC technique or X-ray microtomography (Brodribb *et al.*, 2017; Pratt *et al.*, 2019; Venturas *et al.*, 2019; Gauthey *et al.*, 2020; Feng *et al.*, 2023), while a direct comparison of OV and UAE is still missing. We thus aimed to quantify vulnerability to drought-induced xylem embolism using OV and UAE and to directly compare the two methods by performing simultaneous measurements on the same branches. The study was conducted on three conifers (*Picea abies*, *Pinus sylvestris*, and *Pinus cembra*), and results were compared with vulnerability curves obtained using the FC technique on branches collected from the same individuals. We expected good agreement between UAE and FC measurements and differences in vulnerability thresholds between UAE and OV, depending on the xylem depth observed by the OV method.

## Materials and methods

### Plant material

All measurements were performed on branches of three conifer tree species: *P. abies* (L.) H. Karst., *P. sylvestris* L., and *P. cembra* L. For the first two species, plant material was collected from a forest site near Innsbruck (47°16’N, 11°22’E at 650 m a.s.l.), and for *P. cembra* from a site located near Praxmar (47°09’N, 11°07’E at 1800 m a.s.l.) (both sites in Tyrol, Austria).

Between March and April 2023, at least four 1 m long branches (collected from different individuals) per method and species (Table 1) were cut, wrapped in dark plastic bags containing damp paper towels, and transported to the laboratory. Branches were re-cut under water and rehydrated overnight in a water-filled bucket, covered with a dark plastic bag.

**Table 1.** Vulnerability thresholds and their 95% confidence intervals (CIs) of branches of *P. abies*, *P. sylvestris*, and *P. cembra*

Species	Technique	Ψ_12_ (CI 2.5, 97.5%), MPa	Ψ_50_ (CI 2.5, 97.5%), MPa	Ψ_88_ (CI 2.5, 97.5%), MPa	*n*
*P. abies*	OV	–2.65 (–2.45, –2.86) a	–3.41 (–3.31, –3.51) a	–4.02 (–3.88, –4.17) a	7
	UAE	–3.18 (–3.04, –3.34) b	–3.84 (–3.76, –3.93) b	–4.36 (–4.24, –4.49) b	7
	FC	–3.28 (–3.21, –3.34) b	–3.94 (–3.91, –3.98) b	–4.46 (–4.37, –4.54) b	4
*P. sylvestris*	OV	–1.96 (–1.83, 2.12) a	–2.47 (–2.38, –2.56) a	–2.87 (–2.63, –2.96) a	7
	UAE	–2.71 (–2.62, –2.80) b	–3.38 (–3.31, –3.46) b	–3.92 (–3.77, –4.05) b	6
	FC	–2.84 (–2.75, 2.92) b	–3.37 (–3.31, –3.42) b	–3.77 (–3.68, –3.85) b	4
*P. cembra*	OV	–1.72 (–1.64, –1.80) a	–2.12 (–2.07, –2.16) a	–2.42 (–2.37, –2.47) a	7
	UAE	–2.63 (–2.45, –2.75) b	–2.94 (–2.89, –3.08) b	–3.16 (–3.06, –3.53) b	8
	FC	–3.15 (–3.03, –3.31) c	–3.55 (–3.48, –3.62) c	–3.84 (–3.65, –3.96) c	4

OV, optical vulnerability; UAE, ultrasonic acoustic emission; FC, flow–centrifuge. *n* indicates the number of replicates. For each species, different letters indicate statistically significant differences between Ψ_12_, Ψ_50_, and Ψ_88_ measured with different techniques.

In February 2024, branches 2 m long with a diameter of ~5 cm (five branches from different individuals per species) were collected at the same sites to test light transmission in the xylem.

### Vulnerability analyses

#### Optical vulnerability and acoustic emission

For all species under study, branch vulnerability to drought-induced xylem embolism was tested by simultaneously generating vulnerability curves using the optical vulnerability technique (OV; Brodribb *et al.*, 2016, 2017) and the ultrasonic acoustic emission technique (UAE; e.g. Wolkerstorfer *et al.*, 2012; Cochard *et al.*, 2013; Lamacque *et al.*, 2022) on the same branch. Briefly, fully hydrated branches (*n*=6–8 per species) were prepared by removing a short section of bark (~2 cm long and as wide as the branch diameter: *P. abies* 6.41±0.56, *P. sylvestris* 6.96±0.84, and *P. cembra* 6.28±0.35 mm) to expose two sections of the xylem at ~10 cm apart to then perform optical and acoustic measurements (Supplementary Fig. S1A). The first section was covered with a conductive adhesive gel (Aquasonic Clear; Parker Laboratories Inc., USA) to prevent any further water loss and to improve light transmission. Custom-made clamps (http://www.opensourceov.org) containing an 8-megapixel Raspberry Pi Camera v2 connected to a Raspberry Pi single board computer (both Raspberry Pi Foundation, https://www.raspberrypi.org) and light-emitting diodes (LEDs) were placed with the camera facing the bare xylem. This setup allows images to be captured with sufficient spatial resolution to detect changes in light transmittance or reflectance during embolism formation (e.g. Brodribb *et al.*, 2016). The Raspberry Pi single board computer was set to acquire images every 300 s. The second xylem section was covered with silicon grease to prevent any further water loss and optimize acoustic coupling. A 150 kHz resonance acoustic sensor (R15) connected to a pre-amplifier set to 40 dB (Physical Acoustics, Wolfegg, Germany) was tightly clamped to the debarked section of the branch. UAE were recorded using a PCI-8-based system (PAC Micro-II Express Digital AE System; Physical Acoustics, Wolfegg, Germany) at a threshold of 35 dB, and UAEs were recorded using AEwin software (Mistras Holdings Corp., Princeton, NJ, USA). All samples were dehydrated for up to 10 d (except for *P. cembra*, which required up to 20 d) under plastic bags (to avoid excessive transpiration), and the water potential (Ψ) of end-twigs (three per branch, ~10 cm) was measured at different time intervals with a Scholander apparatus (model 1505D pressure chamber; PMS Instrument, USA). End-twigs were carefully cut without moving branches to avoid artificial ultrasonic signals or changes in camera positions.

For OV, images were downloaded and analyzed using Fiji (a Java-based distribution of ImageJ; Schindelin *et al.*, 2012) following established protocols (https://github.com/OpenSourceOV) that subtract successive images showing contrast changes caused by embolism formation. The percentage of embolized xylem area was determined as:


$$\begin{array}{l} \%\;of\;embolized\;xylem\;area\;=\frac{A_{cum}}{A_{max}}\times 100 \end{array}$$ (1)

where *A*_cum_ is the cumulative area of cavitated xylem at a certain Ψ, and *A*_max_ is the maximum area of cavitated xylem recorded after complete dehydration of each branch. In conifers, acoustic activity generally ceases after complete embolization, and we considered the respective plateau to be *A*_max_ (e.g. Cochard, 1992; Mayr and Rosner, 2011; Unterholzner *et al.*, 2020).

Similarly, the cumulative number of acoustic events (AEs) corresponding to the measured Ψ (AE_cum_) was related to the total number of AEs (AE_max_) obtained during dehydration, as:


$$\begin{array}{l} \%\;of\;cumulative\;AE\;=\frac{AE_{cum}}{AE_{max}}\times 100 \end{array}$$ (2)

#### Flow–centrifuge technique

Vulnerability analyses using the FC technique (Cochard *et al.*, 2005; Beikircher *et al.*, 2010) were performed on four fully hydrated branches per species under study. Briefly, branches were cut under water, debarked at the ends, and recut several times with a sharp wood-carving knife (Wheeler *et al.*, 2013) until a 27.4 cm long segment was obtained. Samples were then placed into a 280 mm rotor of a modified Sorvall RC-5 centrifuge (Thermo Fischer Scientific, USA) with both ends in a reservoir filled with distilled, filtered (0.22 µm), and degassed water containing 0.005% (v/v) ‘Micropur Forte MF 1000F’ (Katadyn Products, Switzerland). The temperature in the centrifuge chamber was set at 10 °C. After 20 min of equilibration at –0.25 MPa, the maximum conductivity (*k*_max_) of the stem segment was measured after 2 min stabilization at –0.5 MPa (Christensen-Dalsgaard and Tyree, 2013, 2014; Feng *et al.*, 2015). A vulnerability curve was then obtained by repeated specific hydraulic conductivity (*k*_s_) measurements with a stepwise increase in rpm at intervals of 0.5 MPa, subjecting the stem segment to decreasing xylem pressure until the samples were fully embolized (at least 95–98%). The percentage loss of conductivity (PLC) was calculated as:


$$\begin{array}{l} PLC=(1-\frac{k_{s}}{k_{max}})\times 100 \end{array}$$ (3)

### Xylem light transmission

For the three conifers under study, we also tested the light transmission properties of their xylem by measuring the amount of light passing through progressively thicker xylem sections (Supplementary Fig. S1B, C). Briefly, debarked wood blocks (4×2×2 cm) were prepared from branches of ~5 cm in diameter and 6 cm in length (Supplementary Fig. S1B), which were previously vacuum infiltrated with the same water used for FC measurements for at least 24 h. A 7.4 µm thick longitudinal xylem section was cut using a microtome (Sledge Microtome G.S.L. 1, Schenkung Dapples, Switzerland). To test the actual thickness of these sections, images of 15 longitudinal xylem sections were taken using a light microscope (Olympus BX 41, System Microscope, Austria) connected to a digital microscope camera (ProgRes CT3, Jenoptik, Germany), and their thickness was measured using ImageJ 1.54 software (National Institutes of Health, Bethesda, MD, USA). The average thickness was 7.35±0.35 µm; however, for simplicity, we refer to them as 7.4 µm thick.

A first 7.4 µm thick longitudinal xylem section was clamped with two paper clips to a Cavicam with the light on, and the illuminance of the light passing through was measured using a smartphone with the Light Meter App LM-3000 (Lightray Innovation GmbH, Switzerland) (Supplementary Fig. S1C). Further 7 µm thick longitudinal xylem sections were added one at a time (avoiding air entrapment between sections), and the light intensity was recorded until no illumination was measured. The same procedure was repeated for debarked wood blocks (2×4×2 cm) previously dehydrated to 75% of their full weight (*n*=5).

### Construction of vulnerability curves and statistical analysis

Branch Ψ was coupled with the corresponding relative cumulative embolized area, cumulative number of AEs, or PLC, and vulnerability curves were then fitted to a Weibull function using the R package fitplc (Duursma and Choat, 2017). Thresholds of interest (Ψ_12_, Ψ_50_, and Ψ_88_; i.e. water potential corresponding to 12, 50, and 88% embolism, respectively) and their 95% confidence intervals were extracted from the vulnerability curves using a standard profiling method.

For the xylem light transmission, xylem thickness (µm) was coupled with the corresponding illuminance values (lux), and a non-linear curve was fitted using the R package ggplot2, employing the locally weighted scatterplot smoothing (LOESS) method. To compare illuminance between species, 95% confidence intervals were generated and overlaid on the LOESS curve. All statistical data were analyzed with R 4.3.0 (R Core Team, 2017) at a probability level of 5%.

## Results

### Vulnerability analyses

**Fig. 1.**
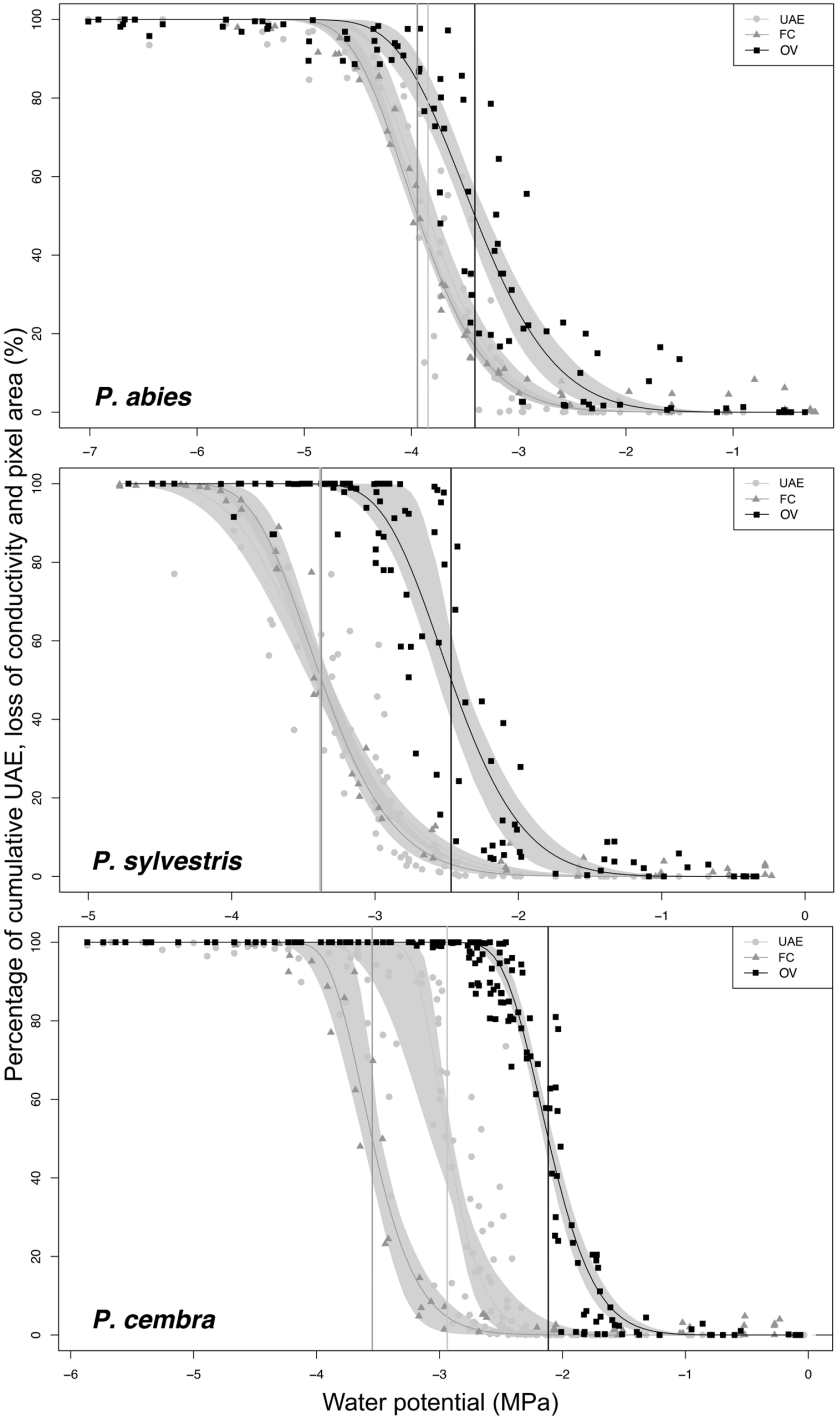
Percentage of cumulative ultrasonic acoustic emissions, loss of conductivity, and loss in pixel area versus water potential. The percentage of cumulative ultrasonic acoustic emissions, loss of conductivity, and loss in pixel area (%) versus water potential (MPa) of branches of *P. abies*, *P. sylvestris*, and *P. cembra* were measured using the ultrasonic acoustic emission (UAE), the flow–centrifuge (FC) and the optical vulnerability (OV) techniques, respectively. Vertical lines represent Ψ_50_. Gray shaded areas represent the 95% bootstrapped confidence interval for fitted curves.

**Fig. 2.**
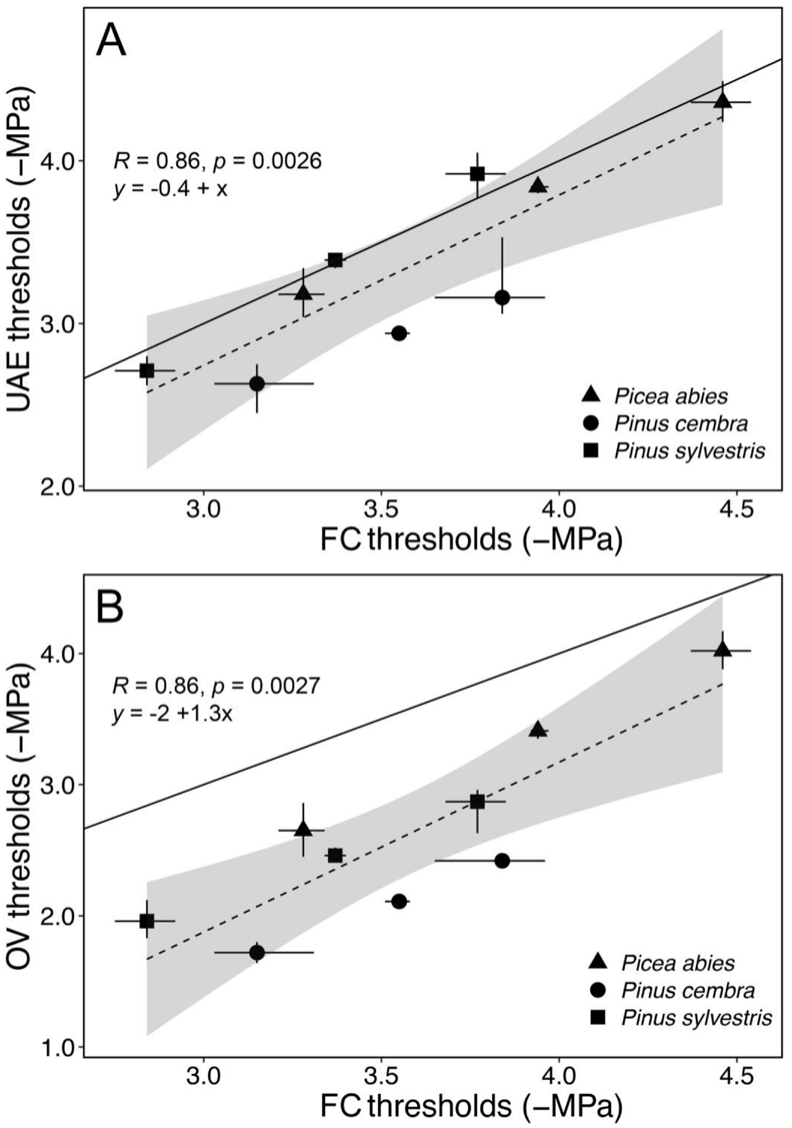
Vulnerability thresholds and their 95% confidence intervals (CIs). The vulnerability thresholds (i.e. Ψ_12_, Ψ_50_, and Ψ_88_) and their 95% CIs were obtained with the ultrasonic acoustic emission (UAE, A) and optical vulnerability (OV, B) techniques versus those obtained with the flow–centrifuge (FC) technique of branches of *P. abies* (triangles), *P. cembra* (squares), and *P. sylvestris* (circles). The dashed line shows linear regression, the solid line is the 1:1 line. Gray shaded areas represent the 95% CIs of the regression lines.

For *P. abies* and *P. sylvestris*, vulnerability curves obtained with the UAE technique did not significantly differ from those obtained with the FC method, though vulnerability thresholds tended to be overall slightly higher (i.e. less negative) with UAE (Table 1; Figs 1, 2A). In *P. cembra*, all UAE vulnerability thresholds (Ψ_12_ –2.63, Ψ_50_ –2.94, and Ψ_88_ –3.16 MPa) were significantly higher than those obtained with the FC method (Ψ_12_ –3.15, Ψ_50_ –3.55, and Ψ_88_ –3.84 MPa) (Table 1; Figs 1, 2A). OV vulnerability curves consistently differed from those obtained with the other two methods, with the respective vulnerability thresholds being significantly higher in all three species under study (e.g. OV versus FC ΔΨ_50_: *P. abies* 0.53 MPa; *P. sylvestris* 0.90 MPa; *P. cembra* 1.43 MPa) (Table 1; Figs 1, 2B).

Overall, conifers under study exhibited species-specific differences in vulnerability to drought-induced embolism, with *P. abies* (e.g. FC Ψ_50_ –3.94 MPa) being more resistant than the two *Pinus* spp. (FC Ψ_50_*P. sylvestris* –3.37 MPa, *P. cembra* –3.55 MPa) (Fig. 1; Table 1).

### Xylem light transmission

**Fig. 3.**
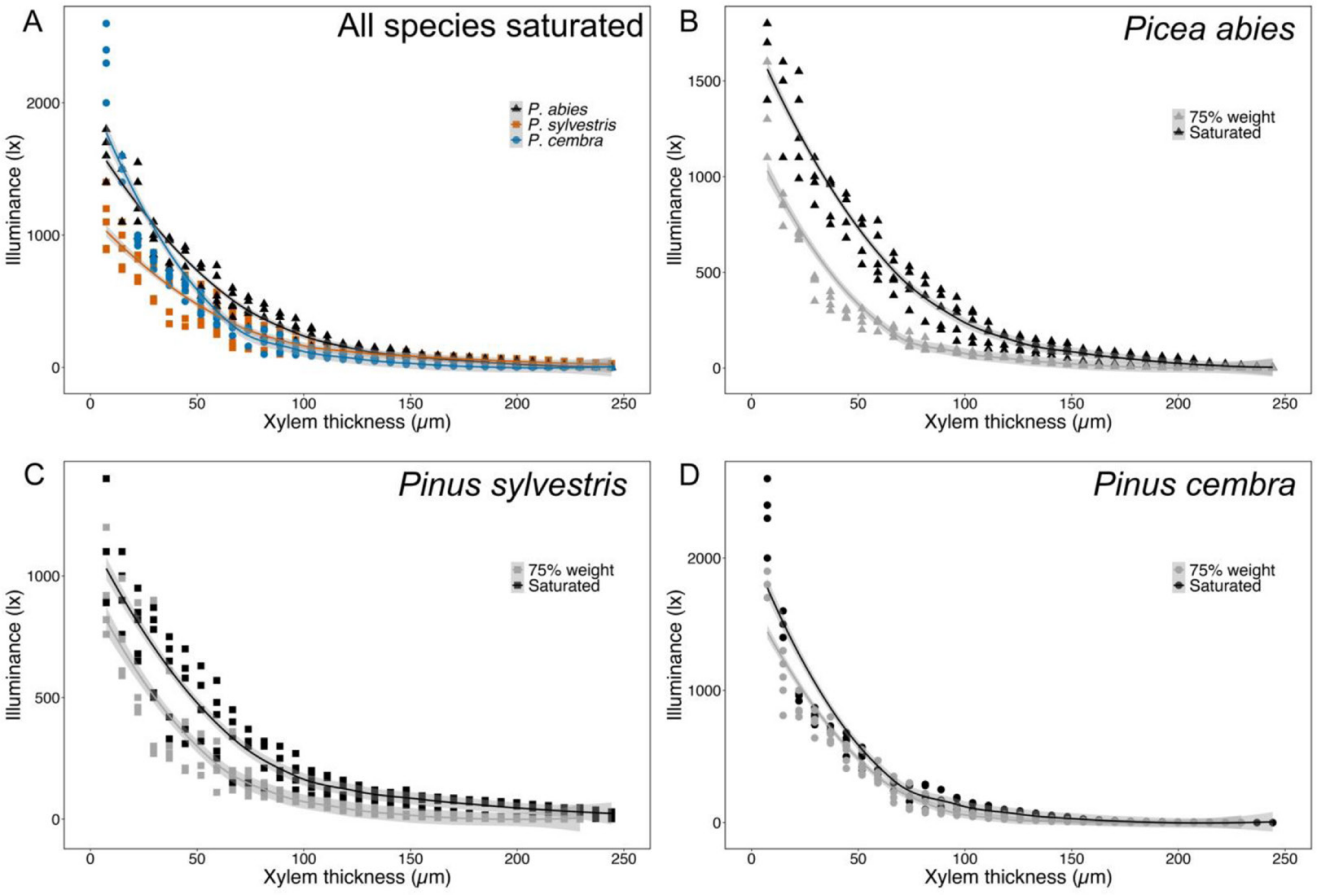
Illuminance measured through different xylem thicknesses. (A) Illuminance (lux) was measured through different xylem thicknesses (µm) of fully saturated branch sections collected from *P. abies* (black triangles), *P. sylvestris* (orange squares), and *P. cembra* (blue circles) (A), and of either fully saturated (black) or dehydrated to 75% of full weight (gray) of branch sections of *P. abies* (B; triangles), *P. sylvestris* (C; squares), and *P. cembra* (D; circles). Solid lines show the mean and shaded areas represent the corresponding 95% confidence interval.

For all species, high illuminance values (~1000–2500 lx) were recorded passing through the first 15–25 µm of xylem, which progressively decreased to values close to zero at xylem thicknesses of ~100–150 µm (Fig. 3A). *Picea abies* and *P. cembra* showed higher initial values of illuminance (1620±130 lx and 2340±192 lx, respectively) than *P. sylvestris* (1098±188 lx). The illuminance in *P. sylvestris* was also lower at increasing xylem thickness, while the illuminance in *P. cembra* xylem dropped from values higher than those of *P. abies* to consistently lower values at ~20–30 µm of xylem thickness (Fig. 3A).

For all three species, the illuminance measured through sections of dried xylem was significantly lower than that measured through sections of saturated xylem (Fig. 3B–D). In progressively thicker dry xylem, illuminance followed similar trends to those observed in saturated xylem, but reached values close to zero at lower thicknesses (90 µm versus 140 µm in *P. abies*, 70 µm versus 130 µm in *P. sylvestris*, and 80 µm versus 105 µm in *P. cembra*) (Fig. 3B–D).

## Discussion

In this study, vulnerability thresholds obtained using the OV technique were consistently higher than those observed with the hydraulic (FC) or the UAE techniques. FC and UAE methods produced overall similar vulnerability curves, except for *P. cembra*, which showed higher UAE thresholds. Higher vulnerability thresholds in OV measurements are probably related to the limited light transmission of the xylem, thus reflecting the hydraulics of only the peripheral xylem layers, or due to an increased xylem surface area exposed to air, which could increase the risk of air seeding. Light transmission in the xylem showed species specificity, with the xylem of *Pinus* spp. transmitting less light than the xylem of *P. abies*. Drier xylem also transmitted less light than saturated xylem, which may affect vulnerability analyses as fewer xylem layers will be observed with decreasing water potential.

For two of the three species under study, vulnerability thresholds did not differ between FC and UAE, indicating agreement between the two methods (Table 1; Figs 1, 2). Ψ_50_ was overall similar to values reported in a previous study performed on plant material collected from the same sites (*P. abies* –3.63±0.04 MPa, *P. sylvestris* –3.58±0.05 MPa; Feng *et al.*, 2021) (see also Table 1). For *P. cembra*, UAE vulnerability thresholds were ~0.6 MPa higher than those obtained with the FC technique (Fig. 1; Table 1). FC Ψ_50_ (–3.55 MPa) was similar to those reported in two previous studies performed on plant material collected from the same site (–3.73±0.07 MPa, Losso *et al.*, 2016; –3.58±0.07 MPa, Feng *et al.*, 2021). The discrepancy between the two methods could be caused by the longer dehydration times required for *P. cembra* (up to 20 d) due to its considerably thicker bark and cuticle compared with the other two species. For species characterized by more effective transpiration protection, such as *P. cembra* (e.g. Anfodillo *et al.*, 2002; Mayr *et al.*, 2003b), bark removal would expose the xylem to faster dehydration rates than the rest of the branch, which may induce artificial acoustic signals near the UAE sensor. We thus recommend future studies to test whether better protection of the exposed xylem (e.g. renewing the silicon grease every few days, maintaining a higher moisture with wet paper towels) could reduce this effect.

Interestingly, OV vulnerability curves were consistently shifted towards higher Ψ than those obtained with the other two methods (Fig. 1; Table 1), thus indicating higher vulnerability to drought-induced xylem embolism. These results disagree with previous studies on conifer branches reporting overall similar results between vulnerability curves generated with the optical and FC techniques (Brodribb *et al.*, 2017; Gauthey *et al.*, 2020). This could be explained by the difference in the size of the samples used, as Brodribb *et al.* (2017) selected smaller branch diameters (3–6 mm) for OV measurements compared with those used for the FC technique. Hence, in smaller branches, OV measurements on peripheral xylem layers seem to be more representative for the overall vulnerability. In our study, larger samples (5–8 mm in diameter) were selected to allow direct comparison with the 27.4 cm long segments required for FC measurements. Furthermore, species-specific differences may play a role, as none of the 14 conifer species analyzed by Brodribb *et al.* (2017) was in the *Pinaceae* family. Although not reported as significant, Feng *et al.* (2023) observed a discrepancy similar to our results for *Pinus halepensis* branches, with OV Ψ_50_ (–3.81±0.14 MPa) being higher than Ψ_50_ (–4.16±0.11 MPa) measured using the FC technique. This study also used smaller diameters for OV measurements (4–5 mm). However, studies comparing OV with other methods in conifers are scarce and may require more attention.

Based on our results, embolism appears to be initiated at less negative water potentials in the outer and younger xylem layers while inner layers are more resistant. We are not aware of any studies that have investigated radial patterns of embolism formation in the three species under study, but previous studies on other conifer species (Choat *et al.*, 2015; Miller *et al.*, 2020) have reported that embolism tends to occur in the inner xylem at lower water potentials than in the younger outer xylem due to a hydraulically safer anatomy. However, embolism patterns may be complicated by differences between earlywood and latewood and between reaction and opposite wood (Spicer and Gartner, 1998; Mayr and Cochard, 2003; Rehschuh *et al.*, 2020). Debarking may influence embolism patterns by increasing the area of xylem in direct contact with air and thus at risk of air seeding (Sperry and Saliendra, 1994; Mayr *et al.*, 2006; Venturas *et al.*, 2019). Further studies in conifers, especially with *in vivo* visualization by X-ray microtomography, may shed light on the propagation of xylem embolisms in debarked stem sections following OV protocols.

In angiosperms, previous interpretations of observed discrepancies between OV and other standard methods, such as benchtop dehydration and X-ray microtomography, suggested that partial debarking of stems could lead to the induction of artificial air seeding (Venturas *et al.*, 2019), thus creating a new starting point for embolism events and consequent shifting of the vulnerability curve (Hacke *et al.*, 2022). However, if this were the case, UAE vulnerability curves should also show a general shift towards higher values as the method requires a preparation similar to OV. This was not the case, suggesting that removing the bark but applying hydrogel to reduce evaporative water loss has a negligible effect in most species (Johnson *et al.*, 2020). It should be noted that less gel was applied for OV (to ensure the visibility of xylem tissue), which may explain the observed shifts in vulnerability in OV but not UAE (where thick gel layers were used). Another main difference between the two methods is that UAE has a greater spatial sensitivity than OV, as acoustic signals can be detected several centimeters away from the acoustic sensor, whereas the OV technique is limited to the field of view of the camera and the transmission/reflectance of the light in the xylem. Accordingly, in a recent study comparing micro-computed tomography and OV, Johnson *et al.* (2020) found that the OV method reliably captured xylem embolism events only to a depth of 0.3 mm into the stem of two Australian angiosperm species. In our study, we also tested the light transmission properties of the xylem by measuring the amount of light passing through progressively thicker xylem sections for all three species under study and found light to reach values close to zero already at depths of 0.15–0.20 mm (Fig. 3). It should be noted that our approach did not consider light reflectance, which is critical for image acquisition while using the OV technique on branches, and thus may even have overestimated the maximum depth at which xylem embolism could be reliably captured. The shallower depths recorded for conifers (i.e. <0.3 mm, equivalent of 20–38 tracheid rows for the species under study; based on Mayr and Cochard, 2003; Mayr *et al.*, 2003a; Martínez-Vilalta *et al.*, 2009; Losso *et al.*, 2016) can be attributed to their different anatomy, as denser wood and the presence of resin and resin channels may limit light transmission. Given the reported shallow depth of field when using the OV method in conifers, additional caution must be exercised when measuring during xylogenesis as vulnerability thresholds of immature and mature xylem may differ.

Light was transmitted differently in the three species under study, with *P. abies* and *P. cembra* having higher initial illuminance values (i.e. at thinner xylem thicknesses) than *P. sylvestris* (Fig. 3A), which had mainly lower illuminance values than the other two species. *Pinus sylvestris* is known for its hard and dense wood, which may limit light transmission. The illuminance of the xylem of *P. cembra* dropped from values higher than those of *P. abies* to consistently lower values at ~20–30 µm of xylem thickness (Fig. 3A). The more abundant resin channels and overall thicker tracheid walls in *Pinus* spp. (Losso *et al.*, 2018) may account for the observed differences in light transmission. This species specificity in light transmission corresponds to the observed shifts in vulnerability curves, as differences between FC and OV curves were least pronounced in *P. abies*, where light entered the xylem deeper than in *Pinus* spp. (Fig. 1; Table 1). Overall, the OV method shows vulnerabilities of the most peripheral xylem layers, while the other two methods used in this study analyze the main vulnerability of entire samples. In dehydrated xylem (i.e. wood dehydrated to 75% of full weight), light transmission decreased in all three species (Fig. 3B, C). This is important because it indicates that the xylem portion, where embolism formation can be observed by OV, decreases during dehydration and thus may affect OV measurements. Hence, as dehydrated wood allows less light to pass through, the xylem region observed by the camera is increasingly restricted to layers close to the surface (i.e. the youngest cell layers).

In conclusion, the higher vulnerability thresholds of the three conifers under study, measured using the OV technique, were mainly caused by the methodical protocol followed (i.e. using the same sample size used for FC and UAE). Compared with the other two methods, OV thus showed a different aspect (i.e. xylem near the surface) of the average vulnerability of the branches, and depended on the species and the size of the organ under study. As the outermost xylem layers are the newest and functionally most important (depending on the time of the year measurements are performed), OV can provide a targeted method for the hydraulic analysis of the latest tree rings. We believe that the OV method, as a highly accessible and affordable method, is a powerful tool for investigating xylem embolism and obtaining vulnerability curves, provided that precautions in data interpretation are taken. This is particularly relevant for conifers, as their peculiar anatomy and physiology limit light transmission and reflectance at very shallow xylem depths.

## Supplementary data

The following supplementary data are available at *JXB* online.

Fig. S1. An ultrasonic acoustic emission sensor and a Cavicam installed on a branch of *P. cembra* for vulnerability analyses, and a schematic representation of the experimental setup used to test for light absorption in xylem sections of *P. abies*, *P. sylvestris*, and *P. cembra* wood.

eraf075_suppl_Supplementary_Figure_S1

## Data Availability

The data underlying this article will be shared on reasonable request to the corresponding author.
