# Supplementary material for: Optical observations of embolism in three conifers overestimate the vulnerability of stem xylem to hydraulic dysfunction
Source: J Exp Bot. 2025 Feb 28;76(10):2864–73. doi: 10.1093/jxb/eraf075 (PMC12223502; doi:10.1093/jxb/eraf075)
Supplement: eraf075_suppl_Supplementary_Figure_S1 [file eraf075_suppl_supplementary_figure_s1.pdf]

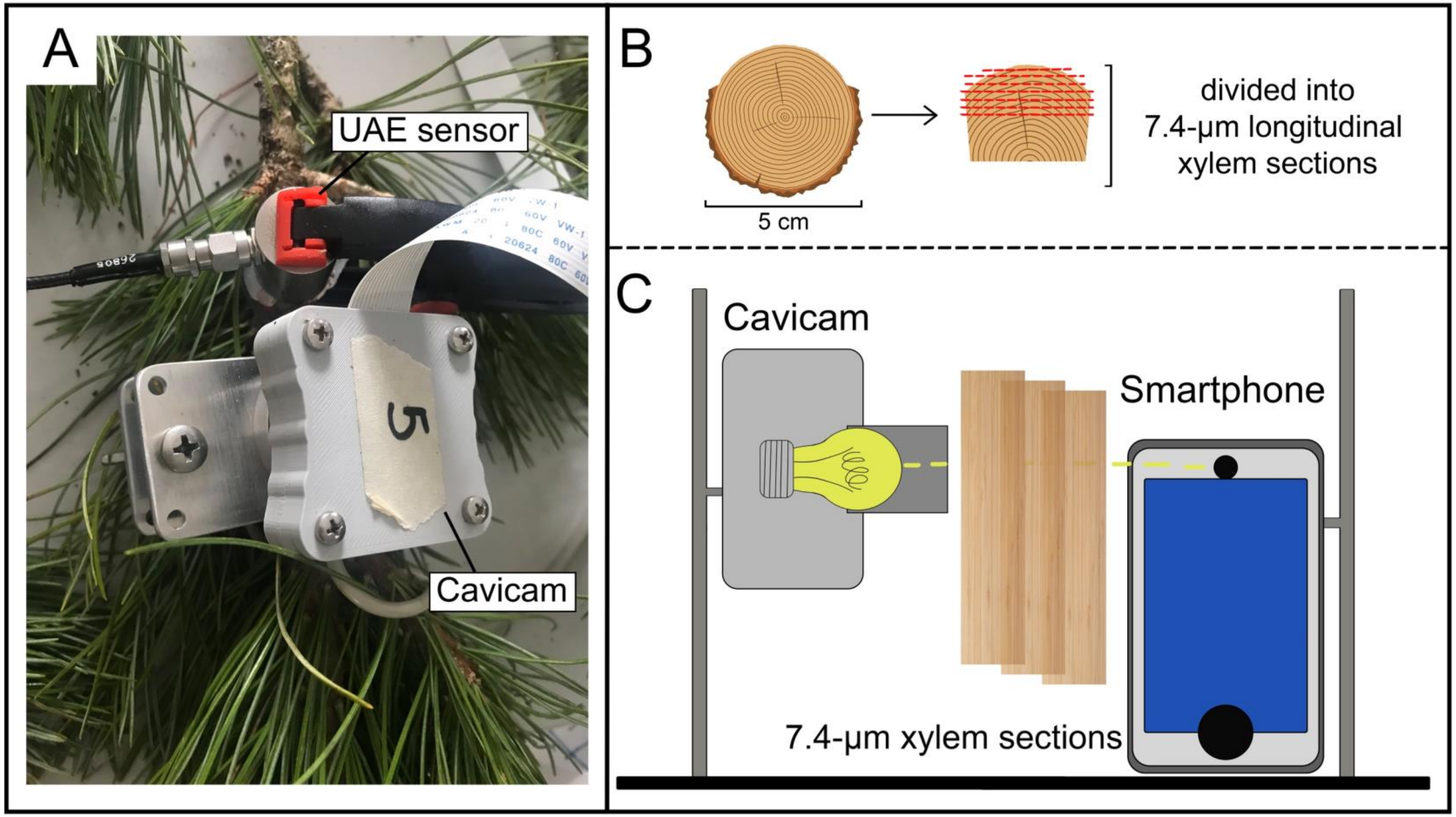

Fig. S1. An ultrasonic acoustic emission (UAE) sensor and a Cavicam installed on a branch of *P. cembra* (A) for vulnerability analyses, and a schematic representation of the experimental setup used to test for light absorption in xylem sections (7.4 µm each) of *P. abies*, *P. sylvestris* and *P. cembra* wood (B-C). A branch section (5 cm diameter) was prepared to fit in a microtome to cut 7.4-µm xylem sections (red dashed lines) (B). 7.4-µm xylem sections were then clamped one by one to a Cavicam with the light on (C). The illuminance of the transmitted light was measured using a smartphone with the Light Meter App LM-3000.
